# Modelling Carbon Emissions in *Calluna vulgaris*–Dominated Ecosystems when Prescribed Burning and Wildfires Interact

**DOI:** 10.1371/journal.pone.0167137

**Published:** 2016-11-23

**Authors:** Victor M. Santana, Josu G. Alday, HyoHyeMi Lee, Katherine A. Allen, Rob H. Marrs

**Affiliations:** 1 Department of Evolutionary Biology, Ecology and Environmental Sciences, University of Barcelona, Barcelona, Spain; 2 Department of Crop and Forest Sciences-AGROTECNIO Center, University of Lleida, Lleida, Spain; 3 Team of Environmental Impact Assessment, National Institute of Ecology, Seocheon, Republic of Korea; 4 School of Environmental Sciences, University of Liverpool, Liverpool, United Kingdom; Argonne National Laboratory, UNITED STATES

## Abstract

A present challenge in fire ecology is to optimize management techniques so that ecological services are maximized and C emissions minimized. Here, we modeled the effects of different prescribed-burning rotation intervals and wildfires on carbon emissions (present and future) in British moorlands. Biomass-accumulation curves from four *Calluna*-dominated ecosystems along a north-south gradient in Great Britain were calculated and used within a matrix-model based on Markov Chains to calculate above-ground biomass-loads and annual C emissions under different prescribed-burning rotation intervals. Additionally, we assessed the interaction of these parameters with a decreasing wildfire return intervals. We observed that litter accumulation patterns varied between sites. Northern sites (colder and wetter) accumulated lower amounts of litter with time than southern sites (hotter and drier). The accumulation patterns of the living vegetation dominated by *Calluna* were determined by site-specific conditions. The optimal prescribed-burning rotation interval for minimizing annual carbon emissions also differed between sites: the optimal rotation interval for northern sites was between 30 and 50 years, whereas for southern sites a hump-backed relationship was found with the optimal interval either between 8 to 10 years or between 30 to 50 years. Increasing wildfire frequency interacted with prescribed-burning rotation intervals by both increasing C emissions and modifying the optimum prescribed-burning interval for minimum C emission. This highlights the importance of studying site-specific biomass accumulation patterns with respect to environmental conditions for identifying suitable fire-rotation intervals to minimize C emissions.

## Introduction

The ability to control carbon (C) budgets at both global- and regional-scales is a key step in tackling anthropogenically-driven climate change [1]. In fire-prone ecosystems, the balance between C fixed in vegetation and that emitted through burning biomass will determine whether a particular ecosystem is a net source or sink for C [2–3]. It is well known that at the global-scale wildfires in fire-prone ecosystems release significant amounts of C annually [4]. On the other hand, prescribed fire is also a significant C source, but it is commonly used as management tool for minimizing wildfire hazard, maintaining habitat quality, creating new agricultural land and stimulating pasture and forest regeneration [5–7]. An obvious, yet ambitious, challenge for ecologists is, therefore, to optimize prescribed fire management techniques to maximize provision of required ecological services and minimize C emissions [6, 8–9]. Whilst C emissions are a global problem, management solutions must be locally-based and dependent on the specific characteristics of each ecosystem [10–12]. The failure to develop regional management plans, holistically-coordinated with the aim of reducing C emissions and increasing C fixation will slow efforts for tackling climate change at the global scale [1, 8].

Undoubtedly, the potential of a given ecosystem to release C by combustion will, at least in part, be determined by the amount of available above-ground biomass–i.e. the fuel load [12–13]. Where there are no constraints on plant productivity, for example by fire or grazing animals, the above-ground biomass of terrestrial vegetation is determined largely by climate (temperature and rainfall), but modified locally by soil-type, land management and historic land use [14–15]. Such gradients of biomass production are clearly defined worldwide, and embrace scales ranging from local ecosystems to biomes [14, 16]. Indeed, ecosystem properties that control biomass accumulation, such as net primary productivity and decomposition rates, are linked closely to climate conditions that vary along both temperature and moisture gradients [14, 17–18]. It is, therefore, important to determine the relationship between biomass-production gradients and C emission patterns. Fire activity at global- and regional-scales is linked to these gradients [19], and it will provide evidence-based information to establish reliable policies for minimizing C emissions along the gradients [20].

Apart from the available above-ground biomass, fire regime and its fluctuations are important factors controlling C emissions in any given ecosystem. The fire-return interval, for example, defines the accumulated amount of biomass burned within a period of time, which is clearly a function of the ecosystem regeneration capacity through time [7, 12]. Similarly, fire severity (i.e. the amount of organic matter consumed by fire) [21] is also important in determining the combustion completeness (CC) in any given fire event. For example, differences in CC between wildfires and prescribed fires should be expected on average, with greater amounts being lost under wildfire conditions. Normally, wildfires occur within fire-prone days (i.e., dry and hot conditions), and can produce the devastation of large areas and high CC; in some cases the fire can burn into the underlying soil organic layers, increasing the amount of C lost [22]. In contrast, prescribed fires should be only performed under controlled climatic conditions, so that undesirable escape fires are avoided, and CC should be much lower [12, 22]. Determining the effects of fire regime variations on C emissions is, therefore, fundamental to understand C budgeting and to design appropriate management systems. Increasing our knowledge in this area is crucial because of global climate change; forecasts for the next few decades predict shifts in wildfire regimes in many fire-prone ecosystems worldwide through increasing dry, hot summer climates with an obvious predicted increase in wildfire frequency, area burned and CC [23].

Excellent examples of fire-prone ecosystems that are traditionally-managed by prescribed burning are moorlands and heathlands dominated by the dwarf shrub *Calluna vulgaris* (L.) Hull (hereafter *Calluna*). These ecosystems are dominant in many parts of Great Britain, but extensive areas are also found throughout northern Europe [24]. In Britain, prescribed fire has been used for centuries for promoting sheep grazing and red grouse *Lagopus lagopus scoticus* (Latham) for sporting purposes [25]. These moorlands are now also required to provide a range of ecosystem services ranging from biodiversity, the provision of potable water and C storage [5, 26–27], and there are continuing pressures to prevent or reduce the use of prescribed burning for moorland management [28].

At present, any reduction in biomass (fuel load) may act to protect these ecosystems against wildfires by minimising fire likelihood and burn severity. However, this reduction has reduction been mainly brought as result of management for other activities (e.g., grazing or hunting). Over the next century, the role of prescribed burning to reduce fuel loads may be necessary to mitigate the effects of climate change [28].

In Great Britain, land managers can apply rotational prescribed burning to moorland during a defined winter burning season (October to mid-April) within a licensed framework [29](example for England). Specifically, prescribed burning should only be done when climatic conditions are optimal for minimizing fire escapes and ecosystem damage. However, there has been increasing controversy in the last years about the optimal fire rotation interval [26–27]. The current recommendations in England and Wales are that rotations should be no less than 10 years [30], whereas in Scotland recommendations are to burn only heather taller than 20 cm [31]. The actual rotation interval, in contrast, is closer to 20 years in England [32] and may be as much as 50–100 years in Scotland [33]. In terms of C storage, there is no a clear management recommendation for *Calluna*-dominated ecosystems. Some practitioners suggest that prescribed burning should be halted altogether [34]. Allen et al. [12], on the other hand, recommended a rotation interval either of short duration (8–12 years) or long duration (>25 years) for a test moorland in central England, but an avoidance of intermediate durations.

One of the major difficulties in defining generalised management prescriptions is the wide differences in growth rates and biomass loads within Great Britain [7]. Clearly, any rotation interval has a potential associated annual carbon loss, which depends on the interaction between the biomass accumulated between burns and the fire-return interval. It is, therefore, important to develop site-specific management plans which reflect site biomass accumulation when developing methods for reducing C emissions. In this assessment, however, it is essential to take into account all the emissions produced by prescribed burning but also to account for C emissions from wildfire, which occur sporadically on British moorlands and take many decades to recover [22]. To investigate this, Allen et al. [12] using a modelling approach at a single site suggested that by modifying the prescribed-burning rotation interval, C emissions from potential wildfire could be minimized. Further assessments of the interaction between prescribed burning rotation interval and wildfire using multiple contrasting sites are needed for predicting future emissions scenarios, especially as wildfire frequency is predicted to increase as a consequence of ongoing global climate change [23, 35–36].

Here, we assess C emissions resulting from different prescribed-burning rotation intervals at sites with differing biomass accumulation patterns. We used biomass-load accumulation data from four *Calluna*-dominated ecosystems along a north-south gradient in Great Britain. The matrix-model based on Markov Chains developed in Allen et al. [12] was then used to predict (i) above-ground biomass-loads, and (ii) annual C emissions; both under different prescribed-burning rotation intervals. This allowed us to assess the optimal prescribed burning interval where C loss is minimized, considering that prescribed burning cannot be halted altogether because it should be used for promoting other ecosystem services (e.g., promoting biodiversity, grazing and hunting). Additionally, in order to assess the impact of future climate change scenarios, we assessed the effects of (iii) increasing combustion completeness (CC) on its own, and (iv) interactions with a variable wildfire return interval (every 50, 100 and 200 years). Estimates of peat accumulation or loss were not included in our models because prescribed burning is mainly performed in winter, when soils are wet or frozen, and therefore, the impact of this management actions on peat layer are assumed negligible [5]. Thus, this work was only focused on the assessment of C emissions of the above-ground biomass loads and its related component fractions (i.e., *Calluna* and litter). The outcomes of C emission simulations are fundamental to develop site-specific management plans for reducing C emissions. At first, we fitted site-specific above-ground biomass accumulation curves for biomass load comparisons, and then we tested the following hypotheses relating prescribed burning and wildfires:

- Hypothesis 1: The optimal prescribed-burning rotation interval, (i.e., the point at which annual C loss is minimized) will be controlled by the different site-specific, above-ground biomass accumulation patterns.
- Hypothesis 2: When prescribed-burning intervals interact with different wildfire return intervals, the optimal prescribed-burning rotations where annual C loss is minimized are altered.

## Methods

### Site descriptions

**Fig 1:**
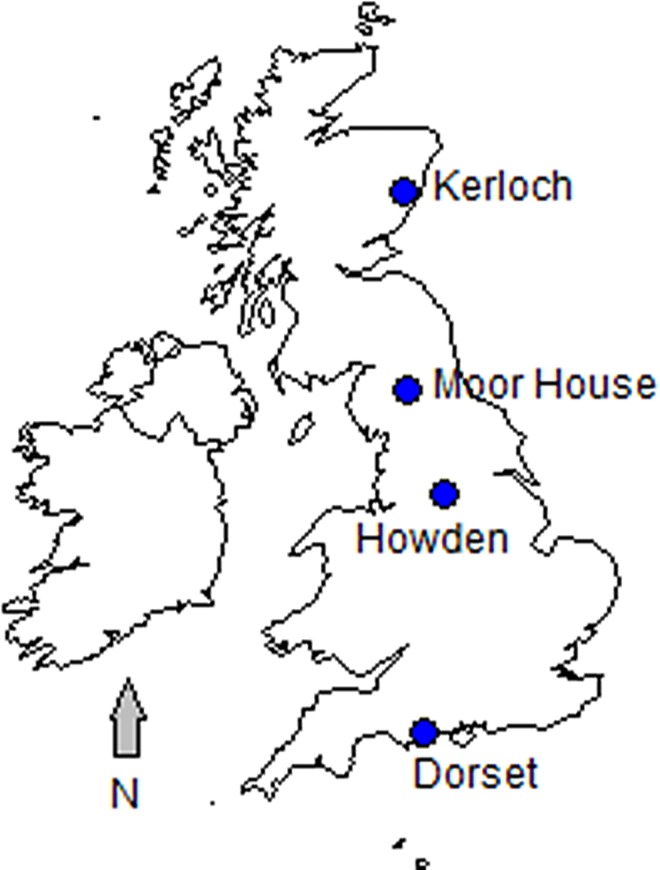
Locations of the four heath/moorland study sites in Great Britain. Geographic coordinates for Kerloch: 56°58’N, 2°30’W; Moor House: 54°41’N, 2°24’W; Howden: 53°28’N, 1°42’W; and Dorset: 50°43’N, 2°07’W.

Biomass accumulation was measured in four contrasting heathlands/moorlands dominated by *Calluna vulgaris* located on a north to south transect running through Great Britain of *ca*. 700 km (Fig 1). Kerloch, the most northerly site, is in Kincardineshire, north-east Scotland (altitude range = 140–280 m). Two sites, Moor House and Howden, are located at opposite ends of the Pennines (Fig 1). Moor House is at the northern end in Cumbria (altitude range = 600–650 m), whereas Howden is at the southern end in the Peak District National Park, Derbyshire (altitude range = 272–540 m). Finally, biomass data were available from three southern sites (Hartland Moor, Studland Heath and Morden Bog), which are fairly close together in Dorset; all at low altitude (≤15 m).

In general, the climate is oceanic but there is a considerable gradient from north to south, also influenced by the altitude. Kerloch is the coldest site with an annual mean temperature of 7.3°C, followed by Moor House (8.1°C), Howden (9°C), and Dorset, the warmest site, with an annual mean temperature of 10.4°C. Annual rainfall, however, does not follow the temperature pattern; Moor House experiences the highest precipitation (1314 mm), followed by Kerloch (1040 mm), Howden (829 mm) and Dorset (800 mm). The four contrasting moorlands also differed in soil types. The vegetation at both Moor House and Howden is on Blanket Bog (peat > 50 cm); the underlying bedrock at Moor House comprises a series of almost horizontal beds of limestone, sandstone and shale [37], whereas at Howden it is a mixture of mudstone, siltstone and sandstone [38]. Soils at Kerloch, in contrast, are poorly-drained, peaty podzols derived from granite and granitic gneiss [39], whereas Dorset soils are podzols of low fertility derived from Eocene deposits (Bagshot Sands) [40].

Heathlands and moorlands are managed in three ways across this latitudinal gradient: (1) two of the upland sites (Howden and Kerloch) were managed by rotational prescribed burning to increase sheep and/or red grouse production. Moor House has been managed actively in the past, but over the last 60 years only a small-scale experiment designed to test different burning rotations (and used here) is still burned. In Dorset, the vegetation management is such that vegetation is allowed to go through the *Calluna* four-phase, growth-cycle defined by Watt [41–42], although the cycle is interrupted frequently by wildfire. The diverse climatic and management conditions combine to produce different plant communities, albeit all dominated by *Calluna*. See S1 Appendix for a more detailed description of vegetation.

### Biomass assessment: collection and derivation

Experimental treatments performed allowed us to collect space-for-time substitution data on changes in biomass (litter and *Calluna*) at each site, encompassing the main growth-phase-cycles of *Calluna* development (from 2 to 50 years). Data on biomass accumulation at Kerloch and Dorset was abstracted from previously-published literature (Kerloch: [39]; Dorset: [43]) where assessments were performed in a co-ordinated way within the International Biological Program [44]. In contrast, data from Moor House and Howden were obtained from experimental surveys (Moor House: [7]; Howden: [12]; data available in the University of Liverpool data catalogue, DOI: 10.17638/datacat.liverpool.ac.uk/58.). A detailed description of monitoring and surveys carried out in each site are detailed in S1 Appendix.

### Statistical fitting of biomass accumulation curves

At Kerloch and Dorset there was an initial increase in both *Calluna* and litter biomass which stabilised with time towards an asymptote. Non-linear Gompertz curves[y~ae^-be*exp(-cx)^] were fitted to these data by the authors [39, 43]. Following this premise, Gompertz curves were also fitted here, after testing it was the best fit. At Howden, data showed a linear increase of *Calluna* and litter biomass with time, but inspection of residuals and Q-Q plots indicated heteroscedasticity. Biomass and time data were thus log_e_ transformed in order to meet homoscedasticity requirements [12]. In the case of Moor House, *Calluna* and litter variables where modelled similarly to Kerloch and Dorset, using Gompertz curves. The models were fitted using non-linear mixed-effects models to account for spatial pseudo-replications [45]; time was used as fixed factor and grazing-burning treatments nested within blocks as random factors. All regression models were fitted within the R Statistical Environment [46].

### Modelling biomass accumulation and carbon release

The impact of prescribed-burning rotation interval on (i) above-ground biomass and (ii) annual C released was modelled using the algorithm developed in the R Statistical Environment [46] by Allen et al. [12], where full details and the code are provided. The model is based on a Markov Chain or Leslie matrix [47], and the first stage of this model creates a predicted long-term, stable, age-structure of moorland/heathland vegetation under varying prescribed-burning rotations. Over time, this model tends towards a stable age distribution, which can be used to make predictions about the population in the long-term. For these calculations the model assumes that the first age at which the vegetation can be subject to prescribed burning is eight years. Here, all rotation intervals ranging from 8 to 50 years were tested, because this is the range over which field data were available for all four sites. In addition, an asymptotic relationship with little further above-ground fuel-load accumulation was observed for all but one site (Howden) studied after 25–30 years. The proportion of total area burned in each step (annual area burned), was calculated as 1/rotation interval. To achieve this, all areas 8 years and older were burned with probability 1/(rotation interval–years not burned) [12].

Once the stable age-structure was created for a given rotation interval, the associated biomass-load was calculated using the derived regressions relationships through time of *Calluna* and litter (described in the previous section; Table 1). For this, a bootstrapping procedure was used which multiplied the proportion of moorland in each age-class by a random draw of the predicted distribution of biomass-load. These calculations were repeated 10,000 times to give the estimated mean and 95% confidence limits. The sum of both fractions across all age-classes gives the long-term amount of above-ground biomass-load. Biomass values were calculated in tonnes per hectare (t ha^-1^). Similarly, the mass of above-ground carbon (C_mass_) was estimated by a further bootstrapping procedure multiplying predicted biomass-load and a random draw of measured carbon concentrations (derived from a Peak District study: 48.3 ± 0.1% for *Calluna* and 49.0 ± 0.1% for litter) [12].

**Table 1:** Parameters of the selected models for biomass accumulation patterns through time since the last burning (years). Data from the selected four sites in Great Britain was modelled independently: (a) *Calluna* biomass (t ha^-1^) and (b) litter biomass (t ha^-1^).

	(A) *Calluna* biomass					(B) Litter				
Site	Model selected		a	b	c	Model selected		a	b	c
Kerloch	Gompertz	Estimate	22.96	3.22	0.88	Gompertz	Estimate	20.65	2.61	0.89
		SE	0.89	0.46	0.01		SE	0.79	0.31	0.01
		t value	25.62	6.97	67.09		t value	26.12	8.21	72.49
		*P*	<0.001	<0.001	<0.001		*P*	<0.001	<0.001	<0.001
Moor House	Gompertz	Estimate	7.94	8.07	0.78	Gompertz	Estimate	8.90	1.70	0.70
		SE	0.80	3.55	0.04		SE	0.70	10.83	0.90
		t value	10.08	2.28	20.12		t value	12.65	0.16	0.77
		*P*	<0.001	0.035	<0.001		*P*	<0.001	0.877	0.045
Howden	Linear [log (y) ~ log (x+1)]	Estimate	-0.93	1.15	-	Linear [log (y) ~ log (x+1)]	Estimate	3.86	1.1	-
		SE	0.05	0.02	-		SE	0.04	0.02	-
		t value	-19.98	48.14	-		t value	92.61	51.32	-
		*P*	<0.001	<0.001	-		*P*	<0.001	<0.001	-
Dorset	Gompertz	Estimate	20.15	3.58	0.86	Gompertz	Estimate	28.38	8.67	0.86
		SE	0.62	0.53	0.01		SE	2.96	6.11	0.04
		t value	32.41	6.75	59.79		t value	9.56	1.42	20.47
		*P*	<0.001	<0.001	<0.001		*P*	<0.001	0.173	<0.001

The annual C released by prescribed-burning (C_lossPBA_) at each rotation interval was then estimated as the product of the annual area burned (calculated as 1/rotation interval) [12], a random draw from the C_mass_ distribution, both for the given rotation interval, and a random draw from the combustion completeness (CC) distribution (Hypothesis 1). CC used was calculated from prescribed fires set at Howden data (71.4 ± 2.6% for *Calluna* and 54.5 ± 2.8% for litter) [12], since similar data were not available for the other sites and site effect in CC is not expected (i.e., similar vegetation). In addition, in order to assess the effect of increasing CC in C_lossPBA_, we ran the model for the different sites with CC values of 20, 40, 60, 80 and 100%. Calculations were performed bootstrapping each CC with a ±5% error. The 100% CC value is extreme but very intense wildfire in similar systems has shown consumption of the above-ground biomass to be at this level [22]; therefore, its use can give us an idea of the maximum amount of carbon emissions from above-ground biomass produced by intense fire events in these systems.

Finally, long-term predictions of the impact of prescribed burning rotations and superimposed wildfire were calculated over a period of 200 years (C_lossPB200_), i.e. four cycles of 50-years (Hypothesis 2). Three different time periods between the superimposed wildfires were considered (50, 100 or 200 years). Little is known about the wildfire return interval in *Calluna*-dominated vegetation in Great Britain, but a study of peat cores on Robinson’s Moss (Peak District National Park; K. Halsall, personal communication) showed that between 1000 and 3000 years ago wildfires occurred at approximately 125-year intervals [12]. Hence, here we based our 100 and 200 year fire return intervals to straddle this value. The lower wildfire return interval (50 years), was included to assess the effects possible interval shortening as a result of ongoing climate change [48]. We know that wildfire occurrence is stochastic, however, we superimposed our wildfire return intervals in a deterministic manner in order to facilitate comparisons between them and with ordinary deterministic prescribed burning rotations. Here, the model was run initially from post-wildfire conditions, i.e. all vegetation was burned and started in age-class 1(100% of area modelled). Carbon lost in prescribed fires was summed over the time period between wildfires for each rotation interval and added to the predicted value of total carbon mass per hectare (C_mass_). Use of C_mass_ to represent carbon loss in a wildfire assumed maximum biomass-load consumption, i.e. that CC was 100% of all age-classes (including<8 years) [12]. Finally, we also calculated C emissions produced by different wildfire return intervals in the absence of prescribed burning. For this, the C amount after 50 years in the absence of fire was multiplied by the number of fires in 200 years; i.e., four times in a 50 year wildfire return interval, twice in a 100 years return interval, and once in a 200 year return interval.

## Results

### The above-ground biomass accumulation patterns

**Fig 2:**
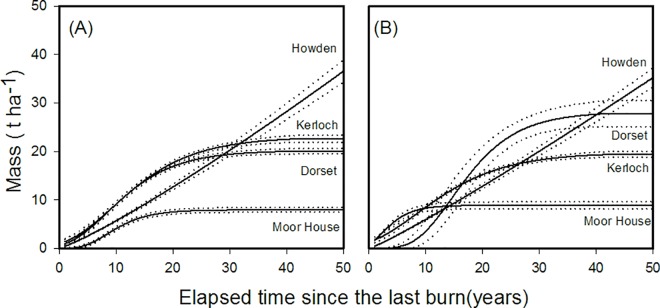
Biomass accumulation curves (solid lines) for the above-ground biomass of (a) *Calluna* and (b) litter depending on the elapsed time since the last burn and for different sites in Great Britain. Dotted lines indicate the standard deviations of the curves.

Above-ground biomass accumulation patterns through time since last burn differed between sites (Fig 2A). These differences, however, were not ordered along the north-south gradient. Moor House, one of the sites with colder temperatures and higher precipitation, had the lowest *Calluna* biomass values, and grew slowly until it reached 20 years after fire with an asymptote around 8 t ha^-1^. Surprisingly, the two sites at the extremes of the climatic gradient (Kerloch and Dorset) showed intermediate and similar accumulations; growth occurred over the first 20 years until an asymptote around 20 t ha^-1^was achieved approximately 25 years after fire. These two sites were also those that regenerated more quickly and reached the greatest biomass values quicker after fire. *Calluna* biomass at Howden, the site ranked as the second warmest and driest (after Dorset) had the greatest biomass, increasing linearly until *ca*. 35 t ha^-1^was measured 50 years after fire.

Accumulation patterns for litter also differed between sites (Fig 2B). Although *Calluna* accumulation data for Kerloch and Dorset were similar, litter showed different responses. Litter accumulated faster at Kerloch in the first few years towards an asymptote at approximately 20 years, whereas in Dorset, litter accumulation followed a clear sigmoidal curve with an early lag-phase (0–10 years), and a phase of rapid increase (10–30 years) before reaching an asymptote around 30 years. The asymptotes for these sites were also different; Dorset reached 29 t ha^-1^ compared to 20 t ha^-1^ at Kerloch. At Howden litter increased linearly until *ca*. 35 t ha^-1^was accumulated 50 years after fire. At Moor House litter accumulated quickly in the first ten years but then reached an asymptote of *ca*. 9 t ha^-1^, and was the site with the lowest litter asymptote (Fig 2B).

**Fig 3:**
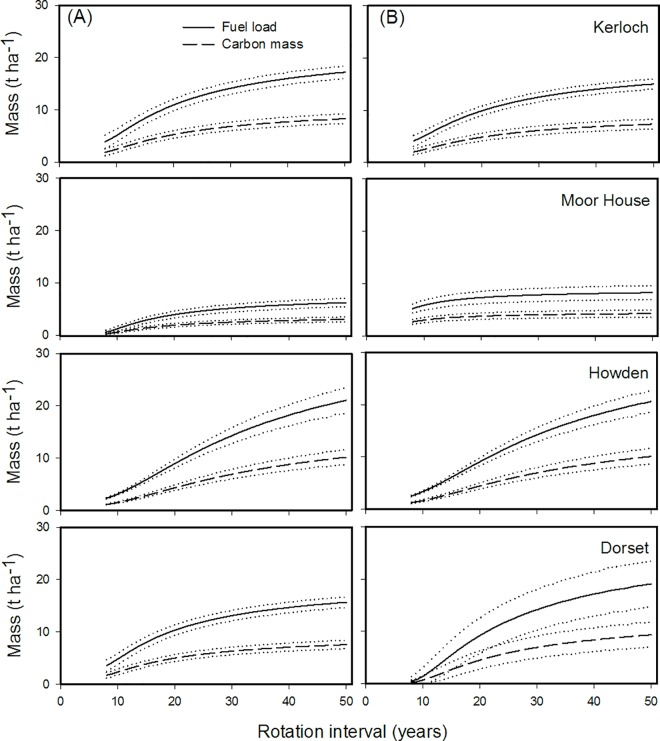
Predicted long-term modelled above-ground biomass load and carbon mass of (a) *Calluna* and (b) litter for four different sites in Great Britain under various rotation intervals. Mean values (solid lines) and 95% confidence limits (dotted lines) from 10,000 bootstrapped values are shown.

Modelling simulations derived from the stable age structure for each prescribed burning interval showed that, the predicted above-ground biomass (and associated C_mass_) increased for both *Calluna* and litter with the prescribed burning interval for all sites (Fig 3). As expected, the greatest biomass loads were found in the sites with the largest biomass accumulation rates; i.e. Howden, followed by Dorset and Kerloch, and Moor House with the lowest value (Fig 3B).

### Hypothesis 1: the optimal prescribed-burning rotation interval will be controlled by the different site-induced patterns of fuel accumulation

**Fig 4:**
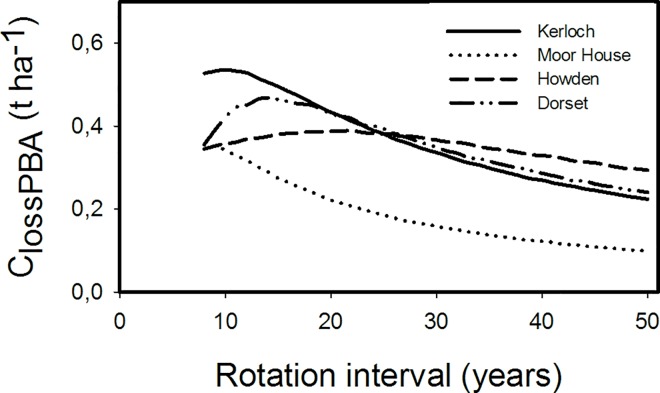
Modelled annual carbon loss due to prescribed burns (C_lossPBA_) for four different sites across Great Britain under varying rotation intervals.

**Fig 5:**
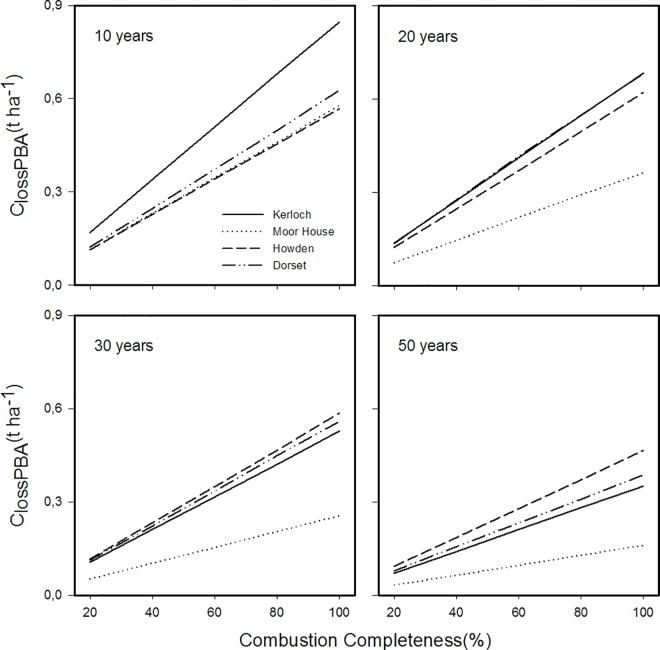
Modelled annual carbon loss at different prescribed burns rotation intervals for four different sites across Great Britain under varying combustion completeness (CC) scenarios.

The annual carbon lost through prescribed burning (C_lossPBA_) was highly variable depending on the site studied (ranging from 0.1 to 0.55 t ha^-1^, Fig 4). Two clear patterns were also detected depending on the climatic conditions of sites. At the sites with the lowest temperatures and highest precipitation (Kerloch and Moor House), short rotation intervals of *ca*. 8–10 years maximized carbon emissions. In contrast, the warmer and drier sites (Dorset and Howden) demonstrated a hump-shaped response with the highest C emissions at intermediate rotation intervals. Emissions were maximized in Dorset at *ca*. 15 year intervals, whereas Howden showed a less pronounced hump-shaped curve with a maximum loss at 15–25 year intervals. Carbon lost was therefore minimized at long rotation intervals (30–50 years) for all sites, but for Howden and Dorset short prescribed-burning rotation intervals (8–10) can also minimize C emissions. As expected, higher combustion completeness (CC) increased the carbon annual loss (C_lossPBA_), especially for sites with faster regeneration after fire (Kerloch and Dorset; Fig 5).

### Hypothesis 2: Wildfire interaction with prescribed-burning rotation interval and its effect on C emissions

**Fig 6:**
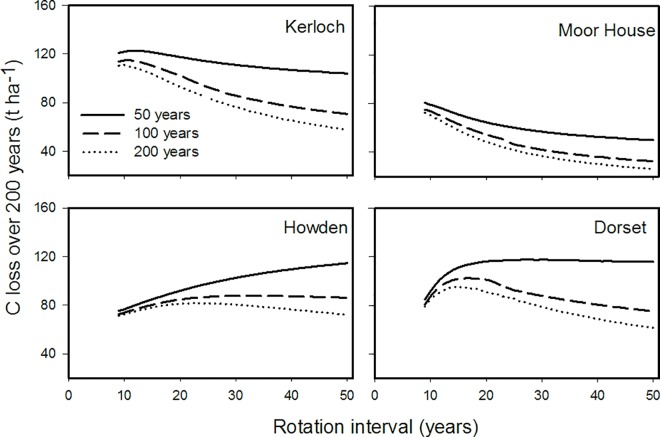
Modelled carbon loss for four sites across Great Britain over a 200-year period with respect to prescribed-burning rotation interval and subjected to an additional wildfire at 50-year, 100-year and 200-year return intervals.

The impact of superimposed wildfires over the prescribed-burning rotations showed that increasing the wildfire frequency increased the carbon loss (C_lossPB200_), at all sites (Fig 6). Moreover, at the two sites with warmer and drier conditions (Howden and Dorset), wildfire frequency also modified the range of prescribed-burning rotation intervals at which C loss was minimized. At Howden, C loss at a 200-year wildfire return interval was minimized with prescribed burnings at short- and long-rotation intervals (8 and 50 years), and greatest emissions were at intermediate rotations (15–25 years). However, shorter wildfire return intervals (50 and 100 years) changed this pattern incrementally. At the 50-year wildfire return interval, C loss increased considerably with lengthening prescribed-burning rotation intervals, the 100 year wildfire return interval produced an intermediate response (Fig 6); at both return intervals the lowest emissions were predicted at an 8 year prescribed burning rotation frequency (Fig 6). In Dorset, C loss at a 200-year wildfire return interval was also minimized with prescribed burnings at short- and long-rotation intervals (8 and 50 years), with maximized emissions at intermediate rotations (13–16 years). The predicted pattern was, however, modified at both 100 and 50-year wildfire return intervals. At the 100-year wildfire return interval, prescribed burning at short- and long-rotation intervals (8 and 50 years) minimized C emissions, and emissions were greatest at prescribed burning rotation intervals between 12 and 22 years. The 50-year wildfire return interval increased C loss at long prescribed-burning intervals, reaching an asymptote of maximized emissions at prescribed-burning intervals between 15 and 20 years (Fig 6).

Emissions at different wildfire return intervals in the absence of prescribed burning had considerable lower values (Table 2). Emissions were between one and four times lower for 50 year wildfire return interval, between 2 and 7 times lower for 100 year return interval, and between 4 and 14 times lower for 200 year return interval.

**Table 2:** C emissions over 200 years (Closs_PB200_) at different wildfire return intervals in the absence of prescribed burning (t ha^-1^).

Site	Wildfire return interval		
	50 years	100 years	200 years
Kerloch	54	27	13
Moorhouse	20	10	5
Howden	90	45	23
Dorset	58	29	15

## Discussion

### The site-induced biomass accumulation patterns

Above-ground *Calluna* biomass accumulation patterns differed between sites as expected, but surprisingly they did not increase along the north to south climatic gradient. It seems that site specific factors, such as soil-type and management, are also important drivers of above-ground biomass patterns. Here, three different responses can be outlined. First, Moor House, with its low mean temperature (8.1°C), highest rainfall (1314 mm) and highest altitude *ca*. 650 m experienced the lowest above-ground biomass accumulation. It appears that the Moor House climate and altitude interact to limit *Calluna* biomass accumulation [7]. Second, Kerloch and Dorset, being the most northern and southern sites respectively, with the lowest and warmest mean temperatures respectively (7.3 and 10.4°C), with contrasting rainfall patterns (1040 and 800 mm), experienced similar and intermediate *Calluna* accumulation rates. The low above-ground biomass production in Dorset has been already attributed to their very low soil fertility [49]. In contrast, it seems that the relative climatic harshness of Kerloch is mainly responsible for the reduced *Calluna* biomass accumulation there [39]. Finally, the central site of Howden, with intermediate temperatures and rainfall (9°C and 829 mm), experienced the largest accumulation after 50 years without fire. It is well known that warmer sites experience conditions for growing more vigorous above ground biomass and reach higher amounts of biomass accumulation at longer times since fire. Moreover, Howden, unlike all other sites is surrounded by large industrial conurbations and the area is well known to be affected by past and current industrial pollution including nitrogen deposition [50]. Here, therefore, growth responses could be expected to be enhanced artificially by nitrogen deposition. For example, Howden has the higher nitrogen deposition (29.96 kg N ha ^-1^ yr^-1^), whereas the other sites have lower and similar values (20.3 for Kerloch, 19.88 for Dorset and 19.46 kg N ha ^-1^ yr^-1^for Moor House; data extracted from www.apis.ac.uk) [51]. In addition, Howden is by far the one that more exceeds the annual critical load (CL) of nitrogen deposition; Howden and Moor House are considered blanket bog systems with CL of 5–10 kg N ha ^-1^ yr^-1^, while Kerloch and Dorset were considered heaths with CL of 10–20 kg N ha ^-1^ yr^-1^. In any case, previous research has suggested a climate-induced biomass gradient in British *Calluna*-dominated ecosystems [18, 49], where annual production is enhanced by high levels of summer sunshine and temperature, and reduced by the number of frost days in the previous winter [17–18]. Our results indicate that though climate is important in determining *Calluna* biomass accumulation, it is not necessarily an over-riding, universal explanatory factor at all sites, and other site-specific factors such as soil fertility, pollutant load, management and altitude (e.g., Moor House) [7] can significantly alter above-ground biomass accumulation patterns. Such site-specific constraints must be considered in future when developing site-specific and national-scale management strategies.

Interestingly, litter accumulation patterns appeared to follow a North-to-South gradient. Kerloch and Moor House experienced the highest accumulations in the first years after fire, but with the passage of time, accumulated litter reached an asymptote much lower than southern warmer sites (Howden and Dorset). It is well known that colder and wetter sites can accumulate higher levels of litter in the first stages after fire because the low intensity of fires (cool burns) reduce combustion completeness [5], and low decomposition rates. In addition, cold winters can freeze and increase the dieback of many *Calluna* leaves, even before reaching senescent states, and increasing subsequently litter accumulation [52]. The linkage between above ground biomass and litter accumulated in this study, with very good relationship between them (P<0.001, r^2^ = 0.81), may explain the higher accumulation of litter at longer times in warmer sites; i.e., places with higher above-ground biomass accumulation produce more litter through time.

Similarly, patterns of biomass accumulation depending on prescribed-burning rotation interval also varied between sites. As expected, long prescribed-burning rotation intervals increased biomass accumulation. These results suggest that a key point in determining the minimum prescribed burning rotation interval that maximizes biomass accumulation is the age at which above-ground biomass reaches its asymptote. It was observed that the later the time taken to reach the asymptote, the longer the prescribed-burning rotation interval. Here, the *Calluna* biomass asymptote for all sites (except Howden) was reached between 20–30 years since the last burn, suggesting that fire-return intervals should be at least as great as the *Calluna* accumulation asymptote (more than 20 years) [7]. However, in the case of Howden, its biomass increased progressively with time since the last burn, and consequently the biomass accumulation depending on the fire rotation interval also followed the same pattern. In any case, these results highlight the importance of studying the biomass accumulation patterns at the individual moorland scale, taking account of site-specific environmental conditions. This will help identify appropriate site-specific fire-rotation intervals, which is fundamental for designing holistic site-specific management plans designed to minimize C loss.

### Hypothesis 1: Annual carbon loss produced by prescribed burning in a particular rotation interval is linked to the pattern of fuel accumulation

Annual carbon loss as function of the prescribed fire rotation interval was also variable depending on biomass accumulation patterns. The colder sites (Kerloch and Moor House), with greater biomass accumulation in the first years (especially litter), experienced greatest annual emissions at short rotation intervals (*ca*. 8–10 years). In contrast, warmer sites (Dorset and Howden) demonstrated a hump-back relationship with largest C emissions at intermediate rotation intervals (*ca*. 15–25 years), and lowest emissions at short- and long- rotation intervals (Fig 4).

C emission behaviour changed with respect to prescribed burning rotation interval across a considerable part of the north-to-south gradient indicating the difficulties of managing sites using simple prescriptions [7, 12]. The same prescribed burning rotation interval may maximize C emission at one site, but be optimal in reducing emissions in another; for example a 10-year rotation interval at Moor House will produce high emissions, but will be optimal to minimize emissions at Howden. This conclusion, therefore, highlights the need for a detailed understanding of biomass accumulation dynamics at the site level to refine burning plans in terms of reducing C emissions. In addition, it is worth noting that the amount of maximum C emitted annually was variable between sites, ranging from 0.38 to 0.53 t ha ^-1^. In this case, higher emission amounts corresponded to sites with fast regeneration immediately after fire (Kerloch and Dorset). Surprisingly, the maximum biomass accumulation reached after a long period without fire seemed unimportant.

Finally, as expected, modelling the impact of changing CC during prescribed burning increased significantly the annual C emitted; reaching a maximum value of 0.85 t ha ^-1^ (CC of 100% at Kerloch with a 10 year rotation interval).This is an increase of between 60 and 123% over our standard model conditions. The greatest increase in C emissions through simulating a higher CC was found in those sites with fastest regeneration after fire (Kerloch and Dorset) and in short prescribed burning rotation intervals (10 years). The implications of these results are worrying because any small increase in CC can increase C emissions, and increased CC is likely under conditions of global warming if prescribed burning has to be done in drier, warmer weather.

### Hypothesis 2: Different wildfire return interval modifies the optimum prescribed burning rotation for reducing annual C loss

Until now we have discussed the relevance of prescribed burning in biomass accumulation and C emitted to the atmosphere. However, it is worth noting that prescribed burning is not the only type of fire in British ecosystems, and wildfires produced by accident and arson occur in spring and summer [52–53]. These wildfires can be very severe and burn significant amounts of above-ground biomass [22]. It is important, therefore, to consider the effects of wildfire superimposed on impacts of prescribed burning when modelling C emissions in future scenarios. Here, we predicted that wildfire would interact with prescribed-burning rotation intervals by both increasing C emissions and modifying the optimum prescribed-burning interval where C emission are minimized. This interaction was also affected by site-specific characteristics and the wildfire return interval. For example, in colder sites, shorter wildfire return intervals (50- and 100-year) only increased carbon emissions. In warmer sites (Howden and Dorset), shorter wildfire return intervals increased C emissions, but also affected the prescribed-burning rotation interval where C emissions were minimized (Table 3). In Howden and Dorset, for example, whereas at 200-year wildfire interval, long prescribed-burning rotation intervals (*ca*. 50 years) minimized C emissions, 50-years wildfire return intervals maximized C emissions at long prescribed fire rotation intervals.

**Table 3:** Optimal prescribed burning rotation interval where C emissions over 200 years (C_lossPB200_) are minimized. These optimal rotation intervals are calculated including the incidence of wildfires at three different return intervals 50, 100 and 200 years.

Wildfire return interval	Site	Optimal prescribed burning rotation interval (years)	C_lossPB200_ (t ha^-1^)
50 years	Kerloch	50	103
	Moor House	50	48
	Howden	8	75
	Dorset	8	85
100 years	Kerloch	50	70
	Moor House	50	33
	Howden	8	72
	Dorset	8 and 50	81 and 75
200 years	Kerloch	50	57
	Moor House	50	26
	Howden	8 and 50	71 and 72
	Dorset	8 and 50	79 and 62

These results, therefore, highlight the uncertainty in establishing fixed prescribed burning rotation intervals at the present time, never mind projecting forward to account for future climate change scenarios or changing wildfire frequency. At present, little is known about the present occurrence of wildfires in Great Britain, but future predictions suggest that these return intervals will be shortened by drier and warmer summers predicted for the future [23, 35–36]. Further studies are sorely needed to assess credible future wildfire regime, because it is a key factor required to design suitable management plans to reduce C emissions in fire-prone ecosystems such as heathlands and moorlands. In addition, the interaction of different wildfire severities with prescribed burning should be included.

### Management implications

Our results provide information to guide policies for the future sustainable management of British and similar European heaths and moors in terms of C budgets. However it is worth noting that recommendations derived here only consider above-ground C balance, impacts on peat and other ecosystem services are not considered. For example, short-rotation prescribed burning programs can promote biodiversity [5, 27]. Moreover, policies must consider wildfire risk and whether prescribed burning has a part to play; i.e., prescribed burning can help to protect against future wildfires by minimising fire likelihood and burn severity.

As noted above, prescribed burning of moorland vegetation is a cultural management practice in Great Britain. The results from this study therefore apply at present just to a limited subset of current vegetation within in the boreal region. However, the modelling approach used and the principles derived and from this study have direct relevance for informing future management of dwarf-shrub vegetation elsewhere in the boreal region. For example, it is predicted that global climate change will produce warmer and drier summers in northern latitudes, and this may result in increased wildfires [36]. In this regard, wildfire extent can be substantive in the boreal region; between 1990 and 1992 for example, large wildfires in Alaska affected 2×10^6^ ha of boreal forests, with many burns occurring over more than 20 000 ha [54]. As a consequence, prescribed burning may be one technique that can be used to minimize damage in protected areas and around human settlements [36], therefore producing scientifically sound management prescriptions based in a clear approach such has been done in this work is fundamental [27].

Irrespective, any policy must take into account site-specific characteristics of biomass production, in this sense, sites with cold and wet conditions, long prescribed-burning rotation intervals (*ca*. every 30–50 years) were optimal for reducing C emissions. In contrast, warmer and drier sites, both short- (*ca*. every 8–10 years) and long- (*ca*. every 30–50 years) rotation intervals were optimal for reducing C emissions; intermediate prescribed burning rotation intervals should be avoided. At the same time, further effort for reducing or increasing prescribed-burning intervals may be needed for mitigate C emissions in some places of England, since the average prescribed-burning rotation interval is at intermediate values and close to 20 years [32]. In contrast, the present management in Scotland may be optimal in terms C budgets, since the average rotation interval is longer, 50–100 years [33]. The management planning for future in British moorlands should also take into account long-term predictions since climate change will increase wildfire frequency [36] and this may exacerbate carbon emissions. If scenarios of warmer and drier conditions occur prescribed burning may only minimize carbon loss if it is applied at short intervals (*ca*. every 8–10 years). In addition, it should be taken into account that in future scenarios of climate change the biomass accumulation patterns will affected by warming. Possible increases in biomass production brought about by warmer, drier conditions should be included in future management plans as it will probably affect C emissions.

## Supporting Information

S1 Appendix Detailed description of plant communities and biomass assessment of study sites. (DOCX)
