# Supplementary material for: Modelling Carbon Emissions in Calluna vulgaris–Dominated Ecosystems when Prescribed Burning and Wildfires Interact
Source: PLoS One. 2016 Nov 23;11(11):e0167137. doi: 10.1371/journal.pone.0167137 (PMC5120849; doi:10.1371/journal.pone.0167137)
Supplement: S1 Appendix — (DOCX) [file pone.0167137.s001.docx]

**S1 appendix.** Detailed description of plant communities and biomass assessment of study sites.

*Plant communities*

The vegetation at Moor House and Howden can be described as *Calluna vulgaris*-*Eriophorum vaginatum* blanket mire (M19) and *Eriophorum vaginatum* blanket and raised mire (M20) communities within the British National Vegetation Classification (NVC; Rodwell 1991). At the Kerloch site, the community was described as a *Calluna vulgaris*-*Erica cinerea* heath (H10) with possible small areas of *Calluna vulgaris-Vaccinium myrtillus* heath (H12). In Dorset, the species list was abstracted from Chapman (1967) and NVC classes fitted using the TABLEFIT program (Hill 2011); the best fit (mean > 66%) community was the *Calluna vulgaris-Ulex minor* heath (H2) community.

*Biomass assessment*

Kerloch biomass was obtained from Miller (1979) where a space-for-time substitution study allowed a reconstruction of biomass accumulation over a 41-year period since burning. Six stands within a 1 km of each other were selected to form a series of increasing age since burning (2, 8, 14, 18, 24 and 36 years). Every stand was sampled annually for a period of six years. Additionally, two stands were specifically burnt and sampled to assess during the early stages of post-fire recovery. For our study, we took the mean values for each stand as presented graphically in Miller (1979) using the online tool provided by the German Astrophysical Virtual Observatory (<http://dc.zah.uni-heidelberg.de/sdexter>, accessed 16 January 2014).

Data for Moor House was described in Alday et al. (2015). The experiment was set up in 1954/5 with four replicate permanent blocks. All blocks were completely burned in 1954/5. Within each block, there were two main-plots to which two grazing treatments (sheep grazing and no sheep grazing) were allocated randomly. Then, within each main-plot, three burning-rotation treatments were also allocated randomly to sub-plots, these were: (i) short-rotation burning (ca. every 10 years), (ii) long-rotation burning (ca. every 20 years), (iii) Not burnt since 1954/5. In addition, each block had an associated unburned reference plot, deemed to have remained unburned for at least ca. 90 years. The Moor House site was sampled in 2011, and the experimental design allowed us to reconstruct biomass accumulation at 5, 16, 56 and 90 years after fire. No effect of grazing in biomass accumulation patterns was found mainly because the sheep grazing pressure was very low (Alday et al. 2015); therefore, we assumed no effect for grazing here.

Biomass accumulation at Howden was described in Allen et al. (2013). Here, a range of stands, previously subjected to prescribed burning, were selected using an age-stratified, random-sampling procedure. The patches were cross-referenced with management maps, providing 22 stands of known ages between 2 and 50 years.

Finally, biomass accumulation data for Dorset was obtained from Chapman et al. (1975). Here, site selection allowed a reconstruction of above-ground biomass over 42 years. Five stands of known age (6, 12, 18, 22 and 36 years) were sampled at two-yearly intervals over a period of six years. Additionally, one stand was specially burnt for this study and was sampled annually for subsequent six years. Here, the mean values per stand as presented graphically in Chapman et al. (1975) were extracted using the procedure outlined above for Kerloch.

The biomass sampling method was similar in all studies. Biomass was harvested from between 3 and 10 quadrats (50 × 50 cm) distributed within the vegetation patches of different ages. All vegetation rooted inside the quadrat was cut at ground level with secateurs. Plant biomass was then sorted into various fractions, usually *Calluna*, other dwarf shrubs, graminoids and bryophytes. In three studies, (Dorset, Howden and Moor House) litter was also collected from the soil surface within the quadrats. Fractions were oven-dried (80^o^C) to estimate the total dry weight per stand. *Calluna* was the dominant species in the vegetation sampled at all sites (80-99%); therefore, for simplicity we only considered the biomass of this species in our analyses and modelling. Bryophytes were a significant part of the biomass at Kerloch (but data not provided) and Moor House (*ca.* 20 % of total biomass), whereas the bryophytes amounts at Howden (<1%) and Dorset (data not provided) were negligible. Because it is expected that bryophyte consumption in fires would be insignificant, this biomass was also not considered in our modelling (Lee et al. 2013). Litter values were not sampled at Kerloch but were estimated here using the linear relationship between litter and *Calluna* biomass derived from the values from the other three sites at sampling square level (y=0.83x+136.26, P<0.001, r^2^=0.809, n=330).

**References**

Alday, J. G., V. M. Santana, H. Lee, K. Allen, and R. H. Marrs, R. H. 2015. Above-ground biomass accumulation patterns in moorlands after prescribed burning and low-intensity grazing. Perspectives in Plant Ecology, Evolution and Systematics 17: 388-396.

Allen, K. A., M. P. K. Harris, and R. H. Marrs. 2013. Matrix modelling of prescribed burning in *Calluna vulgaris*‐dominated moorland: short burning rotations minimize carbon loss at increased wildfire frequencies. Journal of Applied Ecology 50: 614-624.

Chapman, S.B. 1967. Nutrient budgets for a dry heath ecosystem in the south of England. Journal of Ecology 55: 677-89.

Chapman, S.B., J. Hibble, and C. R. Rafarel. 1975. [Litter accumulation under *Calluna vulgaris* on a lowland heathland in Britain](http://apps.webofknowledge.com.ezproxy.liv.ac.uk/full_record.do?product=UA&search_mode=GeneralSearch&qid=1&SID=Z13klfkiv8euvck4cLx&page=1&doc=9&cacheurlFromRightClick=no). Journal of Ecology 63: 259-271.

Hill, M.O. 2011. TABLEFIT: For identification of Vegetation Types (v.1.1). Centre for Ecology and Hydrology, Wallingford, UK.

Lee, H., J. G. Alday, R. J. Rose, J. O’Reilly, and R. H. Marrs. 2013. Long-term effects of rotational prescribed-burning and low-intensity sheep-grazing on blanket-bog plant communities. Journal of Applied Ecology 50: 625-635.

Miller, G.R. 1979. [Quantity and quality of the annual production of shoots and flowers by *Calluna vulgaris* in north-east Scotland](http://apps.webofknowledge.com.ezproxy.liv.ac.uk/full_record.do?product=UA&search_mode=GeneralSearch&qid=1&SID=S2UFDztRo4jsXjw5Vsj&page=1&doc=7&cacheurlFromRightClick=no). Journal of Ecology 67: 109-129.

Rodwell, J.S. 1991. British Plant Communities Vol. 2: Mires and heaths. CUP, Cambridge, UK.
